# IDENTIFICATION OF MUTATIONS IN THE *PAH* GENE IN PKU PATIENTS IN THE STATE OF MATO GROSSO

**DOI:** 10.1590/1984-0462/2020/38/2018351

**Published:** 2020-02-14

**Authors:** Roseli Divino Costa, Bianca Borsatto Galera, Bianca Costa Rezende, Amanda Cristina Venâncio, Marcial Francis Galera

**Affiliations:** a Universidade Federal de Mato Grosso, Cuiabá, MT, Brazil.

**Keywords:** Phenylketonuria, Mutations, Neonatal screening, Fenilcetonúria, Mutações, Triagem neonatal

## Abstract

**Objective:**

To identify phenylalanine hydroxylase (*PAH*) mutations in patients with phenylketonuria (PKU) from the Newborn Screening Service in Mato Grosso, Midwest Brazil.

**Methods:**

This is a cross-sectional descriptive study. The sample consisted of 19 PKU patients diagnosed by newborn screening. Molecular analysis: DNA extraction using the “salting-out” method. Detection of IVS10nt-11G>A, V388M, R261Q, R261X, R252W, and R408W mutations by the restriction fragment length polymorphism (RFLP) technique.

**Results:**

Two mutant alleles were identified in four patients (21.1%), one allele in five patients (26.2%), and none in the remaining ten patients (52.6%). A total of 13/38 alleles were detected, corresponding to 34.2% of the *PAH* alleles present. The most prevalent variant was V388M (13.2% of the alleles), followed by R261Q (10.1%) and IVS10nt-11G>A (7.9%). Three variants (R261X, R252W, and R408W) were not found. The most frequent mutation types were: missense mutation in eight alleles (18.4%) and splicing in four alleles (10.5%). The model proposed by Guldberg to determine a genotype/phenotype correlation was applied to four classical PKU patients with two identified mutations. In three of them, the predicted moderate/moderate or moderate PKU phenotype did not coincide with the actual diagnosis. The prediction coincided with the diagnosis of one classic PKU patient. The estimated incidence of PKU for Mato Grosso, Brazil, was 1:33,342 live births from 2003 to 2015.

**Conclusion:**

The only mutations found in the analyzed samples were the IVS10nt-11G>A, V388M, and R261Q. The genotype/phenotype correlation only occurred in four (5.3%) patients.

## RESUMO

**Objetivo:**

Identificar mutações da fenilalanina hidroxilase (PAH) em pacientes com PKU (fenilcetonúria) do Serviço de Triagem Neonatal em Mato Grosso.

**Métodos:**

Estudo de corte transversal. Amostra composta de 19 pacientes com PKU através do exame de triagem neonatal biológica. Análise molecular: a) extração de DNA pela metodologia “*salting out*”. B) detecção de mutações IVS10nt-11G>A, V388M, R261Q, R261X, R252W e R408W pela técnica de polimorfismo de comprimento de fragmento de restrição (RFLP).

**Resultados:**

Dois alelos foram identificados em quatro pacientes (21,1%), um alelo em cinco pacientes (26,2%) e nenhum nos dez pacientes restantes (52,6%). Um total de 13/38 alelos foram identificados, correspondendo a 34,2% dos alelos PAH presentes. A variante mais prevalente foi a V388M (13,2% dos alelos), seguida de R261Q (10,1%) e IVS10nt-11G>A (7,9%). Três variantes (R261X, R252W e R408W) não foram encontradas. Os tipos de mutações mais frequentes foram: troca de sentido em oito alelos (18,4%) e emenda em quatro alelos (10,5%). O modelo proposto por Guldberg para determinar uma correlação genótipo/fenótipo foi aplicado para quatro pacientes clássicos de PKU, com duas mutações identificadas. Em três, o fenótipo previsto de PKU moderada/moderada ou moderada não coincidiu com o diagnóstico real. A predição coincidiu com o diagnóstico de um paciente PKU clássico. A incidência de PKU estimada para Mato Grosso, Brasil foi de 1:33.342 nascidos vivos para o período de 2003 a 2015.

**Conclusões:**

Foram encontradas apenas as mutações IVS10nt-11G>A, V388M, R261Q nas amostras analisadas. A correlação genótipo/fenótipo ocorreu em quatro (5,3%) pacientes.

## INTRODUCTION

Phenylketonuria (PKU, OMIM 261600)^1^ is an inborn error of metabolism with an autosomal recessive inheritance, predominantly caused by mutations in the phenylalanine hydroxylase (*PAH*) gene. Mutations in the *PAH* gene impair the EC 1.14.16.1 liver enzyme function that catalyzes the conversion of L-phenylalanine (L-Phe) essential amino acids into L-tyrosine (L-Tyr), a precursor of neurotransmitters (dopamine, noradrenaline, and adrenaline) and melanin.^2^ The *PAH* gene is located on chromosome 12 (12q23.2), spanning about 90 kbp in length. The gene contains 13 exons, encoding a 452 amino acid polypeptide, and forming a 1359 base-pair reading transcript. To date, more than 800 mutations have been described in the *PAH* gene.^3^

PKU occurs in all ethnic groups and because of the large genetic variability, prevalence may differ; the highest live birth rates were found in Ireland (1:4,500) and Turkey (1:2,600), and the lowest rates in Finland, Japan, and Thailand (1:200,000, 1:143,000, and 1:212,000 live births).^4^ In Brazil, the incidence varies greatly: Sergipe (1:8,690), São Paulo (1:19,409), Tocantins (1:28,309), Santa Catarina (1:28,862).^5^ In 2012, the prevalence in 18 Brazilian States was 1:24,780 live births.^6^ According to Stranieri,^7^ PKU prevalence in Mato Grosso was 1:33,068 live births in 2009.

Studies have shown substantial variability in *PAH* gene mutations, which are not yet fully known in Brazil. The distribution *PAH* gene mutations among the Brazilian population is highly inconsistent, according to the few studies carried out on the subject. The IVS10nt-11G>A mutation is more frequent in studies conducted in Ribeirão Preto/São Paulo^8^ (17.4%), while an investigation including samples from several States of Northeast Brazil shows a 22.1% incidence rate, and a case study in Alagoas, 53%.^8^ ^,^ ^9^ ^,^ ^10^ I65T mutation was highly prevalent in Rio Grande do Sul (19.5%), a rate similar to that observed in Campinas (17.5%), São Paulo (Southeast Brazil).^11^ ^,^ ^12^ In Minas Gerais, also in the Southeast region of Brazil, the most frequent mutation was V388M, found in 21.2% of the alleles.^13^ V388M (12.7%), R261Q (11.8%), and IVS10-11G>A (10.3%) were the most frequently found mutations in the State of Rio de Janeiro.^14^

Untreated PKU is associated with progressive intellectual disability, accompanied by several additional symptoms, which may include eczematous eruption, autism, seizures, and motor deficits. Developmental problems, aberrant behavior, and psychiatric symptoms often become apparent as the child grows.^15^ The standard treatment for PKU comprises two main joint strategies: a phenylalanine-restricted diet and the use of amino acid-rich metabolic formula, free of phenylalanine.^16^ Thus, the identification of these mutations by molecular study is used as a tool for an accurate PKU diagnosis, guiding the multidisciplinary team towards a more efficient follow-up of the patient.

The objective of this study was to verify the molecular bases responsible for PKU, as well as establish the genotype/phenotype correlation in individuals followed by the Newborn Screening Reference Service at the Júlio Müller University Hospital/Federal University of Mato Grosso (Hospital Universitário Júlio Müller/Universidade Federal do Mato Grosso - HUJM/UFMT) in Mato Grosso (MT).

## METHOD

This is a cross-sectional study. The studied population consisted of patients diagnosed with PKU during the newborn screening at the Newborn Screening Reference Service of Mato Grosso (Serviço de Referência em Triagem Neonatal - SRTN/MT) in the HUJM. The Human Research Ethics Committee of HUJM approved this research under the number 1,486,868, dated April 4, 2016.

The National Newborn Screening Program (Programa Nacional de Triagem Neonatal - PNTN), created by the Ministry of Health/Health Care Office - Regulation ­GM/­MS No. 822, 06/06/2001 -, aims to guarantee not only the screening in this population, that is, tracking diseases included in the National Program, but also to allow children diagnosed with any of these diseases to receive adequate treatment at an early stage, providing an opportunity for proper intervention that can reduce morbidity and mortality. The Ministry of Health (MoH) authorized the State of Mato Grosso in 2001, and UFMT was registered in 2002, through the HUJM, as an SRTN under Regulation SAS/MS No. 684, dated October 4, 2002. In 2002, the service started working on Phase I with congenital hypothyroidism (CH) and phenylketonuria (PKU). Since 2014, SRTN has been working on phase IV of the program (Regulation No. 488, June 17, 2014).

The nominal variables analyzed were age, gender, consanguinity, and family recurrence, along with the following epidemiological variables: PKU incidence in MT and frequency of genotypes and phenotypes. A modified version of the salting-out technique was used to extract the DNA.^17^ The technique described by Saik et al.^18^ was used to perform the polymerase chain reaction (PCR). We investigated the most frequently found mutations in studies carried out in Brazil: IVS10nt-11G>A, V388M (exon 11-primers 5’TGAGAGAAGGGGCACAAATG3’D, 5’GCCAACCACCCACAGATGAG3’R), R261Q, R261X, R252W (exon 7-primers 5’GGTGATGAGCTTTGAGTTTTCTTTC3’D, 5’AGCAAATGAACCCAAACCTC3’R), and R408W (exon 12 - 5’ATGCCACTGAGAACTCTCTT3’D, 5’GATTACTGAGAAACCGAGTGGCCT3’R)^10^ through the restriction fragment length polymorphism (RFLP) technique. Oligonucleotides from GBT Oligos - Custom Oligonucleotide Synthesis Certificate of Analysis, supplied by Ludwig Biotec^®^ (Alvorada-RS/Brazil), were used.

The PCR Master Mix (Ludwig Biotec^®^; Alvorada-RS/Brazil) was used according to the package insert, and the DNA concentration was 100ng/µL. The PCR was performed in the following conditions: initial denaturation at 94°C for five to six minutes, followed by 35 denaturation cycles at 95°C for 30 seconds, annealing at 59°C for one minute, extension at 72°C for 30 seconds, and a final extension at 72°C for five minutes. In the case of exon 11, the annealing temperature was 61°C. For the digestion phase, 5 U of endonucleases were used for all mutations, and the incubation was done overnight at 37°C (Table 1). A 2% agarose gel electrophoresis was used and visualized directly on the Molecular Imager ChemiDoc^TM^ XRS transilluminator and the Image LabTM Software, both from Bio-Rad^®^ (Hercules, California, USA).

**Table 1:** PCR product size in base pairs for exons 7, 11, and 12 of the phenylalanine hydroxylase gene, mutation, endonuclease used, and fragments generated in base pairs.

Exon	PCR product	Mutations	Endonuclease	Fragment(s) after digestion - wild	Fragment(s) (bp) after digestion - mutated
7	263	R261Q ^a^	*Hinf*I	116/147	263
		R252W ^a^	*Aval*	86/177	263
		R261X ^b^	*Ddel*I	263	115/148
11	301	V388M ^a^	*BsaAI*	114/187	301
		IVS10nt-11G>A ^b^	*Ddel*I	301	78/223
12	238	R408W ^b^	*Styl*	238	97/141

^a^destroys restriction site; ^b^creates restriction site.

In order to establish a correlation between genotype and phenotype in PKU patients, Guldberg et al.^19^ created a model in which the sum of the arbitrary values (AV) for each identified mutation would define the clinical phenotype, as shown in Table 1. Thus, clinical cases would be classified as classical PKU, moderate PKU, mild PKU, and non-PKU hyperphenylalaninemia (HPA), according to these values.

The frequencies were calculated as absolute numbers and percentages.

## RESULTS

This study evaluated a total of 19 patients from 15 families. Their follow-up was carried out at the SRTN/MT clinic of the HUJM/UFMT. Patients were distributed in the following age groups: 0-10-year-olds (57.9%), 11-20, (36.8%) and >20 (5.3%). Men added up to 63.2% of the patients, among whom 15.8% showed consanguinity, and 21% were siblings.

The SRTN/MT diagnosed 15 cases of PKU - one case in each of the years 2003, 2005, 2006, 2009, 2013, and 2014; two cases in each of the years 2008, 2010, 2011; and three cases in 2012. Three patients were transferred to Mato Grosso from other SRTNs, and one patient died.

**Figure 1:**
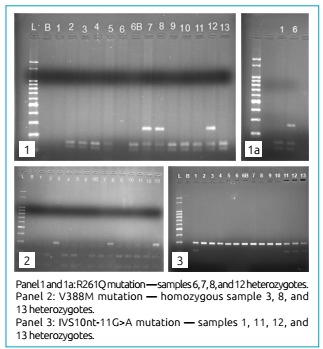
Restriction fragment length polymorphism.

Among the 19 patients participating in the study, four (21.1%) had their two mutant alleles identified. Five patients (26.3%) had only one allele identified, and ten patients (52.6%) remained without identified mutations. The study on the 6 described mutations made it possible to detect 13/38 alleles, corresponding to 34.2% of the PKU alleles of the sample. The most prevalent mutation was V388M (13.2% of the alleles), followed by R261Q (10.1%), and IVS10nt-11-G>A (7.9%). The R261X, R252W, and R408W mutations were not identified. The most frequent mutations were missense mutations, found in eight patients (18.4%), and splicing, found in four patients (10.52%), as presented in Table 2 and Figure 1.

**Table 2:** Genotypes identified in patients with phenylketonuria from the Newborn Screening Reference Service of the State of Mato Grosso.

Patients	Genotypes	Exon	Mutation	Frequency
01	IVS10n-t11G>A	11	Splice	1
03	V388M	11	Missense	2
06	R261Q	7	Missense	1
07	R261Q	7	Missense	1
08	R261Q	7	Missense	1
08	V388M	11	Missense	1
11	IVS10nt-11G>A	11	Splice	1
12	R261Q	7	Missense	1
12	IVS10nt-11G>A	11	Splice	1
13	V388M	11	Missense	1
13	IVS10nt-11G>A	11	Splice	1
14	V388M	11	Missense	1

The genotype/phenotype correlation was based on the multicenter study on phenylalanine hydroxylase deficiency performed by Guldberg et al. in 1998.^19^ This correlation was established from the predicted residual activities induced by each mutation. AVs were determined for each mutation, based on their characteristics: AV=1 - classical PKU mutation; AV=2 - moderate PKU mutation; AV=4 - mild PKU mutation; and VA=8 - non-PKU. The identification of the two mutations produced two AVs, and their sum would define the phenotype predicted by Guldberg et al., creating four clinical variants: classical PKU, moderate PKU, mild PKU, and non-PKU HPA, as shown in Table 1.^12^ We used the first test performed in the newborn screening to apply the classification proposed by Guldberg et al., as seen in Table 3.^19^

**Table 3:** Genotype/phenotype correlation.

Genotype	Phenotype predicted	**Phenotype observed**
V388M	PKU moderate/mild	PKU classical
R261Q/V388M	PKU moderate/mild	PKU classical
R261Q/IVS10nt-11G>A	PKU moderate/classical	PKU classical
V388M/IVS10nt-11G>A	PKU moderate	PKU classical

The incidence of PKU from 2003 to 2015 was 1:33,342 live births in the studied population, according to samples collected at the SRTN. The average coverage of the program in Mato Grosso from 2003 to 2015 was 75.2%, considering 664,943 births in the same period. Also during the same period, five patients were transferred from other Brazilian States.

## DISCUSSION

The most frequently found mutation was V388M, located at exon 11, codon 1162 (c.1162G>A) of the *PAH* gene, in the catalytic domain, consisting of the substitution of the Valine (Val) amino acid for Methionine (Met).^20^ ^,^ ^21^ The V388M mutation was first described in a Japanese patient, is common in the Iberian Peninsula, and has a relatively high frequency in São Paulo and Chile.^20^ It is associated with a severe phenotype and may result from the oxidation of methionine to methionine sulfoxide. Such modification may disrupt the surface of the protein at the interface between subunits and result in the destabilization of the *PAH* tetramer set.^21^ The V388M mutation was the most prevalent in our study, similar to results obtained in the state of Rio de Janeiro,^14^ but different from the data found in studies carried out in Ribeirão Preto/São Paulo^8^ and the South of Brazil,^11^ where the highest prevalence occurred at exon 7.

The R261Q mutation, located at exon 7, consists of replacing the Arginine (Arg) amino acid with Glutamine (Glu) at codon 782 (c.782G>A) of the *PAH* gene, in the catalytic domain. This mutation can be found in several studies published in Brazil, such as those carried out in Ribeirão Preto/São Paulo in 2001 (12.2%), Rio Grande do Sul and Santa Catarina in 2003 (9.8%), Minas Gerais (16.0%), the Northeast, where several States participated (8.7%), and Alagoas (35.0%), but was not identified in the research performed in Campinas/São Paulo in 2008. On the other hand, R261Q was the second (11,8%) most prevalent mutation in the state of Rio de Janeiro.^8^ ^,^ ^9^ ^,^ ^10^ ^,^ ^11^ ^,^ ^12^ ^,^ ^13^ ^,^ ^14^ In a study conducted in the provinces of Qazvin and Zanjan in Iran in 2015, V388M was the second most frequent mutation. This mutation is also common in the Mediterranean and Southern Europe but has a low incidence in Spain.^22^

Most patients in the research study were males (57.9%), similar to the study published in Alagoas.^10^ The prevalent age range was 3-10 years, which had already been predicted, since they were SRTN patients. Patients aged >15 had been transferred from other states. Consanguinity, present in 15.8% of patients, is a deeply rooted social trend in one-fifth of the world population living in the Middle East, West Asia, and North Africa, as well as among migrants from these countries residing in North America, Europe, and Australia.^23^ In Brazil, a cross-sectional epidemiological study using the Key Informants method was conducted in five districts of Rio Grande do Norte, in the Northeast of Brazil, where frequencies of consanguine marriages ranged between 9 and 32%. On average, 25% of consanguineous couples and 12% of non-consanguine ones had one or more children with disabilities. The high prevalence of people with disabilities in the Brazilian Northeast may be associated with the tradition of consanguine marriages in these populations, and some of these deficiencies might result from genetic diseases.^24^ Consanguinity can be measured by the inbreeding coefficient (F). Our study showed marriages between second-degree uncles and nieces and third-degree cousins. The children’s F was 1/8 and 1/16, respectively.^25^

In this study, the presence of the most frequent variants was evaluated according to studies published in Brazil, but only 32% of the mutated alleles were detected.

A study conducted between 2009 and 2014, with families recruited at a genetic counseling clinic in China, selected 118 fetuses from 112 families. The authors identified 63 types of mutations, among which R243Q was the most frequent. Out of the fetuses analyzed, 64 newborns had a normal birth, 31 resulted in abortion, and 23 women were still pregnant at the end of this study. The most prevalent mutation in the abortion cases was R243Q (16.12%), followed by R413P and EXE-96A>G (9.7%), and R261Q (8.1%); our study did not analyze the R243Q mutation, though. The percentage of abortions attributed to the IVS10nt-11G>A mutation was 1.61%.^26^ However, the IVS10nt-11G>A mutation is present in our study, proving that any couple with PKU should have a planned pregnancy with genetic follow-up.

The IVS10nt-11G>A mutation is a type of null mutation located at exon 11 in the intragenic region at codon 1066 (c.1066-11G>A) in the catalytic domain of the *PAH* gene. It was present in this work (7.9%), while in Alagoas, Ribeirão Preto (São Paulo), and the Northeast Region, this mutation was the most prevalent. In Rio de Janeiro, it was the third most prevalent mutation (10.3%),^14^ while being the most frequent one in Portugal and along the Mediterranean coast, which strongly suggests an East-West migration route, even before transoceanic traveling.^9^ Nonetheless, a study carried out with the Iranian population in 2015 did not find the same mutation.

According to the PAHdb,^26^ missense mutations are the most commonly found (60.1%), with deletion mutations holding the second place (10.52%). Our study also showed missense mutations as the most frequent (18.4%), but the second position was held by splice mutations (13.4%).

The genotype/phenotype correlation followed a model proposed by Guldberg et al. in 1998,^19^ which identified two mutations in four patients (21.1%) out of a sample that included 19 individuals. Four patients presented a classical PKU phenotype, but in three of them, the actual phenotype did not agree with what had been predicted, which was moderate/mild or moderate PKU. Thus, agreement between the expected phenotype and the actual one occurred in 1/19 patients (5.3%).^19^

In Alagoas, the genotype/phenotype correlation was performed in 14 individuals out of a sample of 15 patients. Nine patients presented a different genotype from the one expected - instead of moderate/mild PKU, they had classical PKU, similar to our study. In the Northeast, 42.7% (38/89) of the individuals showed mild or moderate PKU, thus establishing a correlation between what was predicted and what was observed.^9^ ^-^ ^10^

A molecular study on tetrahydrobiopterin (BH_4_)-responsive phenylalanine hydroxylase deficiency verified that BH_4_-responsive *PAH* had been recently described as a variant of *PAH* deficiency caused by specific mutations in the *PAH* gene. Studies suggest that the BH_4_ response can be predicted from the corresponding genotypes. Data from BH_4_ loading tests indicated an incidence of BH_4_ in >40% of the general PKU population and >80% of patients with mild PKU. Our study found R261Q and V388M mutations, which are responsive to BH_4_. Regarding IVS10nt-11G>A, its use is not yet clear.^27^

 The PKU incidence in this study was 1:33,342. According to the MoH, PNTN coverage in Brazil was 84% in 2014. The coverage of the screening program in Mato Grosso was below the national rate from 2003 to 2015, but we underline that the exams performed in the private health system are not reported to the screening service. The actions of the ­PNTN/­MT represent a remarkable advance in the secondary prevention of chronic diseases, even though the state coverage is below national levels. Although the SRTN has limitations, such as lack of physical space and qualified professionals, both in the technical and administrative areas, it is considered a good service for Mato Grosso’s society. A negative point is the lack of governmental support both at national and state levels. The MoH should organize national meetings, in which they could discuss the “hits and errors” of each state; there is also a lack of support from the state government, given that they do not provide regular training for the accredited health service in Mato Grosso. In addition, the issues faced by the districts result from a high rate of employee turnover, without giving new workers information about how the SRTN is organized and its importance. Hence, there is an increase in inadequate samples and incomplete forms.

Therefore, it is necessary to create strategies to raise awareness among all those involved, providing continuing education programs, standardizing technical procedures, and understanding the roles played by each one.
